# Development of the opioid self-management scale for advanced Cancer patients with pain and examination of its validity and reliability

**DOI:** 10.1186/s12904-022-00987-4

**Published:** 2022-06-06

**Authors:** Shiori Yoshida, Fumiko Sato, Keita Tagami, Rie Sasaki, Chikako Takahashi, Konosuke Sasaki, Shin Takahashi

**Affiliations:** 1 Department of Oncology Nursing, Health Sciences, Tohoku University Graduate School of Medicine, 2-1 Seiryo-machi, Aoba-ku, Sendai, Miyagi 980-8575 Japan; 2 School of Nursing, Fukushima Medical University, 1 Hikarigaoka, Fukushima, 960-1295 Japan; 3 Department of Palliative Medicine, Tohoku University Graduate School of Medicine, 2-1 Seiryo-machi, Aoba-ku, Sendai, Miyagi 980-8575 Japan; 4 Department of Nursing, Miyagi Cancer Center, 4-7-1 Medeshimashiotenodayama, Natori, Miyagi 981-1239 Japan; 5 Department of Nursing, Tohoku University Hospital, 2-1 Seiryo-machi, Aoba-ku, Sendai, Miyagi 980-8575 Japan; 6 Department of Medical Oncology, Tohoku University Hospital, 2-1 Seiryo-machi, Aoba-ku, Sendai, Miyagi 980-8575 Japan

**Keywords:** Advanced cancer, Cancer pain, Opioid medication, Self-management

## Abstract

**Background:**

Approximately 60% of outpatients with advanced cancer experience pain; therefore, self-management of opioid use is important for appropriate pain relief. To date, no studies have clearly described the concept of opioid self-management or assessed the factors involved, including the improvement of self-management abilities. This study developed, and evaluated the validity and reliability of an opioid self-management scale for advanced cancer patients with pain (OSSA). Opioid self-management in advanced cancer patients with pain was defined as the management of opioid medication performed by patients with advanced cancer to relieve cancer pain on their own.

**Methods:**

Three phases were required for validation and reliability of the OSSA: 1) testing content validity, 2) testing face validity, and 3) testing construct validity, concurrent validity and reliability.

**Results:**

After a three-phase process, the OSSA consisted of 33 items on six subscales. The structural equation modeling was such that the χ^2^ value was 709.8 (*p <* 0.001, df = 467), goodness-of-fit index was 0.78, adjusted goodness-of-fit index was 0.73, root mean squares of approximation was 0.063, and comparative fit index was 0.92. The Pearson correlation coefficients between the total OSSA score and the 24-hour average pain or pain relief over 24 hours were − 0.21 (*p <* 0.05) and 0.26 (*p <* 0.01), respectively. Cronbach’s α was 0.93. The intraclass correlation coefficient range was 0.59–0.90.

**Conclusion:**

The findings of this study show that the OSSA has acceptable validity and reliability, and that better self-management leads to greater pain relief. The OSSA can be considered effective for use in research, but shortened version should be prepared for realistic and practical clinical use.

**Supplementary Information:**

The online version contains supplementary material available at 10.1186/s12904-022-00987-4.

## Background

Approximately 60% of outpatients with advanced cancer experience cancer-related pain, and approximately 20% experience moderate-to-severe pain [1]. Cancer pain is complicated by tumor growth and metastasis, and it reduces patients’ quality of life (QOL). It is presumed that patients have difficulties managing the pain symptoms that they experience outside the hospital.

It is important to relieve total pain; approximately 90% of pain is relieved by pain treatment methods, according to the World Health Organization (WHO) Analgesic Ladder, which focuses on opioid use [2]. However, patients with strong concerns about opioids experience poor pain relief and a poor QOL due to inadequate medication [3, 4]. Patients should reduce their concerns about cancer pain and opioids and consult healthcare providers to receive appropriate opioids for effective cancer pain alleviation.

In Japan, the recommendations for effective pain relief include regular opioid administration, use of rescue medication for breakthrough pain, sufficient countermeasures against side effects, use of appropriate analgesic aids, and patient education; these constitute the WHO cancer pain treatment methods [5]. These support systems are implemented during a patient’s admission, and they include support to increase self-efficacy [6] and self-care abilities [7–11] and reduce concerns about cancer pain and opioids [12, 13]. However, these kinds of support are intended to alleviate cancer pain. To date no studies have clearly described the concept of opioid self-management for advanced cancer patients with pain.

Pain self-management and pain in outpatients with advanced cancer taking opioids have been previously studied [14]. Some elements of these studies were extracted for developing this self-management scale, including pain assessment and adherence to regular opioid dosing, strategies for breakthrough pain, strategies for pain with rescue medication, strategies for opioid anxiety and concern, strategies for pain that cannot be palliative, and strategies for side effects. The results showed that those who could not take opioids in a timely manner had a greater disruption to their lives due to pain. It was observed that advice on medication use by medical professionals can affect a patient’s QOL, and enable patients to have the self-management skills capable of creating an environment in which they can take opioids in their daily life.

To better understand the status of opioid medication management at home, we conducted a qualitative study of home self-management of opioid use for cancer pain relief in advanced cancer patients [15]. The following factors were important: understanding pain and opioids, taking opioids and coping appropriately, coping appropriately to alleviate opioid side effects, having supportive people, and living with the disease. An important factor in the management of opioid medication for pain relief is for patients to reach a psychological adjustment that opioids are part of their life and essential for living with the disease. Previous studies have focused on opioids that are effective for cancer pain, such that patients are relieved from pain and can live their own lifestyle, and identified concepts for enhancing self-management with opioids for patients with cancer [14, 15].

The purpose of this study was to examine the validity and reliability of an opioid self-management scale for advanced cancer patients with pain (OSSA) that was developed based on previous studies. The OSSA is a scale designed for the self-management of opioid medication in advanced cancer patients to help them relieve their cancer pain on their own. The OSSA is expected to help patients understand their way of life through self-management. Improving a patient’s self-management abilities can help them live autonomously, even as their activities of daily living (ADL) decline as their disease progresses. In research, using the OSSA as an evaluation index for developing new care methods enables the measurement of the quality of care provided using the patient self-management approach.

## Methods

The OSSA was developed using a draft scale consisting of items extracted quantitatively [4] and inductively from 10 home-based patients with advanced cancer who were receiving opioids [5]. In addition, some items were extracted deductively from previous studies [14–20]. The validity of the OSSA was assessed using tests for content validity, face validity, construct validity, and concurrent validity. Three phases of validation were required; reliability was examined in Phase 3.

### Phase 1: content validity

Content validity is the assessment of the items of a scale by an expert in the concept [21]. Additional file 1 contains the list of items used for content validity.

#### Participants and procedures

The inclusion criteria were 1) researchers with experience in cancer pain research, and 2) physicians, pharmacists, specialists, and certified oncology nurses with direct involvement in cancer pain care. The exclusion criteria were 1) researchers with no experience in cancer pain research, and 2) physicians, pharmacists, specialists, and certified cancer care nurses with no direct involvement in cancer pain care. The minimum calculated sample size was five [22]; therefore, we aimed to recruit at least five professionals from each category for a total of 30 researchers, physicians, pharmacists, and nurses as participants.

Study information and questionnaire forms were given to the participants between August and October 2018. The variables of interest included occupation, specialist’s qualifications, years of experience in cancer pain research or care, and Draft Scale 1. The scale consisted of 40 items. The evaluation was performed using the content validity ratio (CVR). The CVR is an item statistic that is useful in the rejection or retention of specific items [23]. The participants were asked to choose one of the following responses: 1) *Very appropriate*, 2) *Very appropriate but expression needs to be corrected*, 3) *There is a problem with the appropriateness*, or 4) *Inappropriate*. The CVR was calculated using the formula:

$$\frac{\frac{n_{e}-\frac{N}{2}}{N}}{2}$$

*n*_*e*_ indicates the number of participants who chose “*Very appropriate*” or “*Very appropriate but expression needs to be corrected*.” *N* indicates the total number of participants [24].

The participants who chose “*Very appropriate but expression needs to be corrected*,” “*There is a problem with the appropriateness*,” and/or “*Inappropriate*” were asked to propose corrections or additions and provide feedback.

#### Data analyses

A content validity ratio (CVR) ranges from − 1 to + 1, with a value of 0 indicating that half of the raters needed the item. A high CVR score indicates that it is reasonable for an item to be included in a scale. Lawsh will not adopt an item unless it has a CVR value of ≥0.62 for 10 raters, and the CVR value for adopting an item increases as the number of evaluators decreases [23]. Since more than 10 evaluators were included in this study, it was determined that a CVR value of 0.62 would be sufficient to adopt an item. Proposed corrections and additions to the questionnaire, and feedback provided by the participants were discussed by two nursing researchers with experience in scale development and five graduate students in nursing research. The items were then refined to create Draft Scale 2.

### Phase 2: face validity

Face validity is an assessment of whether the items of a scale are understood easily by an actual group of respondents [25].

#### Participants and procedures

The inclusion criteria were: 1) patients with advanced cancer, 2) administration of opioids for alleviation of cancer pain, 3) > 20 years old, and 4) aware of having advanced cancer. The exclusion criteria were: 1) patients without advanced cancer, 2) administration of opioids for purposes other than alleviation of cancer pain, 3) < 20 years old, 4) aware of having a disease that is not advanced cancer, and 5) having physical, mental, and cognitive disorders. The required sample size was calculated to be 10 [19].

The information document of the study and questionnaire forms were given to the participants, who were outpatients in a designated cancer hospital in October 2018 and patients receiving home-based care. The submission of the questionnaire forms by a participant was considered a consent provision. The assessed variables in the questionnaire were age, sex, performance status (PS), duration of opioid use, and Draft Scale 2. Draft Scale 2 evaluated the participants’ understanding of the questionnaire and solicited suggestions for the improvement of each question item; participants were asked to comment freely.

#### Data analyses

Two nursing researchers with experience in scale development and five graduate students in cancer nursing research discussed the choice of wording for the items, refined the items, and created Draft Scale 3.

### Phase 3: construct validity and concurrent validity and reliability

#### Participants and procedures

Between November 2018 and December 2019, the study information document and questionnaire were distributed to outpatients among three hospitals designated for cancer care. The inclusion and exclusion criteria for Phase 3 were the same as those listed above for Phase 2. The calculated sample size was 130. Based on an α coefficient of 0.90, confidence interval of 0.05, and estimated response rate of 60% [19], 210 participants were recruited. The questionnaires were self-administered and returned on-site or by mail. The submission of a questionnaire form by a patient was considered a consent provision. Patients were retested one week later; these forms were returned by mail.

#### Measures

The validity and reliability of the OSSA variables are shown in Table 1.

**Table 1:** The validity and reliability of the OSSA^a)^ Variables

Scales and Medical Records	Subscales and variables
Participant Characteristics	Age, sex, PS, presence or absence of a caregiver,
	Type of employment, patient history, disease evolution, type of opioids, opioid prescribed and the doses used, duration of opioid use, type of pain and extent of pain (assessed using the numeric rating scale [NRS] for a 24-hour period: worst pain, average pain, pain that interferes with daily life activities and the rate of pain relief).
Self-Care Agency Questionnaire (SCAQ) [26]	Ability to perform self-care operations
	Ability to adjust one’s own physical condition based on personal weaknesses
	Ability to concentrate one’s attention on self-care
	Ability to receive valid support.
*Medication Adherence Scale* [27]	Medication compliance
	Collaboration with healthcare providers
	Willingness to access and use information about medication
	Acceptance to take medication and how taking medication fits patient’s lifestyle

^a)^ Opioid Self-management Scale for Advanced cancer patients with Pain

#### Participant characteristics

Patient characteristics included age, sex, PS, presence or absence of a caregiver, type of employment, patient history, disease evolution, types of opioids prescribed and the doses used, duration of opioid use, type of pain and extent of pain (assessed using a numeric rating scale for a 24-hour period: worst pain, average pain, pain that interferes with daily activities, and the rate of pain relief).

#### Ossa

Draft Scale 3 was used for the Phase 3 testing. Each score is a Likert scale score ranging from 1 point for “*No*” to 5 points for “*Yes*.” Higher scores indicate greater self-management abilities.

#### Self-care agency questionnaire (SCAQ)

The SCAQ has 29 items in four subscales, including 10 items on “*Ability to perform self-care operations*,” 7 items on “*Ability to adjust one’s own physical condition based on personal weaknesses*,” 7 items on “*Ability to concentrate one’s attention on self-care*,” and 5 items on “*Ability to receive valid support*.” The scores range from 1 point for “*No*” to 5 points for “*Yes*.” Higher scores indicate greater self-care abilities [26].

#### Medication adherence scale

The medication adherence scale [27] has 12 items in four subscales, including “*Medication compliance*,” “*Collaboration with healthcare providers*,” “*Willingness to access and use information about medication*,” and “*Acceptance to take medication and how taking medication fits patient’s lifestyle*”; each has three items. The scores range from 1 point for “*Never*” to 5 points for “*Always*,” including two reverse items; a higher score indicates greater medication adherence.

#### Data analyses

All analyses were performed using SPSS version 24.0 for Windows (Japan IBM, Tokyo) and SPSS AMOS version 26.0 (Japan IBM, Tokyo).

Construct validity testing includes exploratory factor analysis (maximum likelihood estimation, Promax rotation, factor loadings greater than 0.45) [17, 28] and confirmatory factor analysis. This study used confirmatory factor analysis to test whether the OSSA data extracted by the exploratory factor analysis validated the factor hypotheses. Other construct validity tests include multitrait scaling analysis [29], and hypothesis testing (hypothesis: “Patients with higher scores on the Opioid Self-Management Scale have lower pain intensity”). Interpretation of the correlation coefficients followed Guilford’s Rule of Thumb [30]. In this interpretation, < 0.2 indicates a slight almost negligible relationship; 0.2–0.4 a low correlation; 0.4–0.7 a moderate correlation; 0.7–0.9 a high correlation, marked relationship; > 0.9 a very high correlation, very dependable relationship.

Concurrent validity was analyzed using the Pearson correlation coefficients between the OSSA, and SCAQ or medication adherence scale. The predictive correlations for concurrent validity are the following: OSSA subscales 1, 2, 3, and 5 concern coping with symptoms, talking to supportive people, and living with acceptance of the illness. We speculated that these are related to the SCAQ subscales concerning self-care operations*,* adjusting physical condition, attention to self-care, and receiving support. However, OSSA subscales 4 and 6 are pain- and opioid-specific and, therefore, not related to the SCAQ. We speculate that OSSA subscales 1, 2, and 3 are associated with compliance with the medication adherence scale, collaborating with healthcare providers, and taking medications according to life. In contrast, we expect OSSA subscales 4, 5, and 6 will not be relevant to the MAS because they are specific to pain, opioids, and disease comorbidity.

Reliability was analyzed using Cronbach’s α coefficient, and intraclass correlation coefficient (ICC) was evaluated by retesting.

## Results

### Phase 1: content validity

#### Sample characteristics

Questionnaires were sent to 67 individuals; 44 persons (65.7%) responded and were included: 9 physicians (20.5%), 32 nurses (72.7%), and 3 pharmacists (6.8%).

#### Content validity

The CVRs for each item of the OSSA are shown in Additional file 1. The CVR range was 0.55–1.00. Four respondents had similar answers that two items, “*I can record the date I used painkillers*” and “*I can record the time I used painkillers*,” are similar and should be combined into one “*date and time*” question; therefore, they were combined. Two items with a CVR of 0.55 (“*I can prepare a generous supply of painkillers*” and “*I can adjust the way I take my painkillers according to the level of pain*”) were deleted. After a discussion on the proposed changes for the expressions used in the items, the data of a woman in her 30s, a woman in her 50s, and a man in his 60s were used to create Draft Scale 2, which comprised 37 items.

### Phase 2: face validity

#### Sample characteristics

Ten persons were invited to participate, and they all responded; 4 (40.0%) were men. The average age (±standard deviation) of the participants was 56.5 ± 10.1 years. The duration of opioid use was 21 ± 32.8 months. The most intense pain was 7.5 ± 2.1, and the average pain was 4.5 ± 1.2.

#### Face validity

The participants responded that 2 of 37 items needed to have their expressions corrected. One of these was “*I can keep as-needed painkillers in an easily accessible place*”; the comments included “*The expression ‘as-needed’ is difficult to understand. ‘Painkillers to take when pain is felt’ is better*.” However, we decided to keep “*as-needed painkillers*” unchanged, as it is a commonly used expression in clinical practice. The second item was “*I can preventively use ‘use-as-needed’ painkillers before pain emerges*,” and participants commented, *“There are times when you don’t know when the pain will emerge, so ‘predictable pain’ would be better*”. We decided to replace it with “*For predictable pain, I can take as-needed painkillers preventively.*” Draft Scale 3 was created after discussing the proposed changes for the expressions.

### Phase 3: construct validity, concurrent validity, and reliability

The questionnaires were distributed to 234 individuals, and 154 (65.8%) provided responses. Of these, we excluded 20 inadequate responses and analyzed the responses from 134 (87.0%) of the forms.

#### Sample characteristics

Table 2 lists the characteristics of the participants involved in the investigation of validity and reliability of the OSSA. Males accounted for 54.5% of the responses, and 63.2% had a PS of 1. Digestive cancer accounted for 56.5% of the cases, and the most common 24-h opioid analgesic prescribed was oxycodone (76.5%).

**Table 2:** Characteristics the participants in the investigation of validity and reliability

characteristics the participants	N		n(%)	Mean	SD
Age	134			59.69	12.7
Sex	134	male	73(54.5)		
		female	61(45.5)		
PS	133	0	11(8.3)		
		1	84(63.2)		
		2	19(14.3)		
		3	19(14.3)		
Caregiver	133	Yes	123(92.5)		
		Unemployed	10(7.5)		
Employment	132	Full-time employee	36(27.3)		
		Part-time or contract employee	15(11.4)		
		Unemployed	81(61.3)		
Site of disease	133	Pancreas	25(18.8)		
		Colon	19(14.3)		
		Lungs	14(10.5)		
		Stomach	13(9.8)		
		Breasts	11(8.3)		
		Liver/gallbladder	9(6.8)		
		Esophagus	9(6.8)		
		Head and neck	3(2.3)		
		Uterus	3(2.3)		
		Prostate	2(1.5)		
		Thymus	2(1.5)		
		Other	23(17.3)		
Type of around-the-clock opioid	132	Oxycodone	101(76.5)		
		Fentanyl	9(6.8)		
		Morphine hydrochloride	1(0.8)		
		Other	21(15.9)		
As-needed opioid	130	Oxycodone	105(80.8)		
		Fentanyl	5(3.8)		
		Morphine hydrochloride	5(3.8)		
		Other	15(11.5)		
Daily morphine equivalent dose	124			48.02	59.55
Duration of opioid analgesics use	132			9.43	19.05

#### Construct validity

In the exploratory factor analysis of the 37-item OSSA, the Kaiser–Meyer–Olkin (KMO) value was 0.851 (*p <* 0.001, Bartlett’s sphericity test). Based on the results of a scree plot, the eigenvalue was set at 6. Four items with a loading factor < 0.45 were deleted, and 33 items were left. With this 33-item OSSA, the KMO value was 0.848 (*p <* 0.001, Bartlett’s sphericity test). Table 3 presents the results of the factor analysis of the 33-item OSSA. As a result, it had six subscales.

**Table 3:** 33-item OSSA^a)^ factor analysis results

Item content	Factor 1	Factor 2	Factor 3	Factor 4	Factor 5	Factor 6
**Subscale 1: Managing opioids and coping with pain (Cronbach’s α = 0.88, ICC = 0.65)**						
No.10 I can take around-the-clock painkillers at a predetermined time.	**0.741**	−0.168	0.030	0.136	0.033	−0.044
No.15 I know whether or not as-needed painkillers are working.	**0.733**	−0.106	0.031	− 0.026	0.055	0.105
No.14 I can quickly take as-needed painkillers as soon as the pain gets worse.	**0.732**	0.097	0.067	−0.091	− 0.053	− 0.047
No.16 When a single dose of as-needed painkillers does not work, I can wait for a certain time before taking the additional drug I have been told to take.	**0.711**	0.114	0.115	−0.108	− 0.162	− 0.078
No.12 I can keep as-needed painkillers in an easily accessible place.	**0.678**	−0.016	− 0.076	0.075	0.112	−0.152
No.13 For predictable pain, I can take as-needed painkillers preventively.	**0.651**	−0.046	−0.024	− 0.071	0.028	0.114
No.8 I know about the side effects of painkillers.	**0.578**	0.093	− 0.051	0.014	−0.042	0.094
No.9 I can make arrangements to ensure that I do not forget to take painkillers (e.g., using a medicine box).	**0.516**	0.033	−0.061	0.084	0.151	0.009
No.7 I know about the effects of painkillers.	**0.469**	0.114	0.031	−0.103	0.032	0.181
**Subscale 2: Talking to health care professionals (Cronbach’s α = 0.93, ICC = 0.62)**						
No.21 I can talk to a medical professional about my anxieties and concerns about painkillers.	−0.030	**0.987**	−0.066	0.004	0.032	−0.005
No.27 I can talk to a medical professional when the side effects of painkillers persist.	−0.167	**0.956**	0.012	0.001	0.064	−0.025
No.5 I can talk to a medical professional about my worries and concerns about pain.	−0.094	**0.756**	0.115	−0.013	0.013	0.143
No.11 I can talk to a medical professional when the effect of around-the-clock painkillers wears off quickly.	0.259	**0.619**	−0.047	0.032	−0.089	0.076
No.17 I can talk to a medical professional when the use of as-needed painkillers does not ease the pain.	0.349	**0.610**	−0.063	0.019	−0.053	− 0.029
No.22 I can talk to a medical professional rather than stopping taking painkillers of my own accord.	0.178	**0.601**	0.010	0.058	0.141	−0.142
No.28 I can talk to a medical professional about my worries and concerns about things other than pain and painkillers.	0.158	**0.498**	0.266	0.057	0.005	−0.057
**Subscale 3: Talking to friends and family (Cronbach’s α = 0.92, ICC = 0.86)**						
No.30 I can talk with family and friends about my anxieties and concerns.	−0.049	− 0.014	**0.996**	0.027	0.007	0.037
No.29 I can talk with family and friends about how to deal with pain.	0.018	0.037	**0.921**	0.000	−0.071	−0.014
No.31 I communicate well with family and friends.	0.096	−0.152	**0.831**	0.092	0.069	−0.041
No.6 I can talk to my family and friends about the severity of pain.	−0.085	0.092	**0.828**	−0.105	0.014	0.079
No.32 I can ask family and friends to help when I am not feeling well.	0.078	0.098	**0.542**	0.017	−0.028	− 0.052
**Subscale 4: Recording pain and opioid use (Cronbach’s α = 0.89, ICC = 0.64)**						
No.18 I can record the date and time I used painkillers.	0.143	−0.293	0.079	**0.886**	0.029	0.022
No.24 I can record the severity and presence/absence of side effects of painkillers (e.g., constipation, drowsiness, nausea, etc.).	−0.104	0.093	0.015	**0.835**	0.006	−0.059
No.20 I can talk to a medical professional based on my management records, such as the severity of pain and the times I took medication.	−0.005	0.203	−0.058	**0.825**	−0.101	0.017
No.19 I can record the number of painkillers remaining.	−0.003	0.043	−0.034	**0.810**	−0.101	−0.062
No.4 I can record the severity of pain in a diary or memo using means such as numbers, a line, or a picture.	−0.141	0.098	0.030	**0.548**	0.151	0.225
**Subscale 5: Living with the disease (Cronbach’s α = 0.86, ICC = 0.69)**						
No.36 I can spend my time feeling calm.	−0.042	−0.015	− 0.082	0.002	**1.028**	0.000
No.35 I can cope with the disease in my own way.	0.034	0.090	−0.052	−0.053	**0.764**	0.025
No.37 I can spend my time in a restful environment.	0.131	−0.034	0.163	0.005	**0.682**	−0.023
No.34 I can carry out activities of daily living (e.g., housework or paid work) in accordance with my physical condition.	0.021	0.118	0.089	−0.046	**0.473**	−0.046
**Subscale 6: Understanding pain characteristics (Cronbach’s α = 0.78, ICC = 0.71)**						
No.2 I know the time intervals between continuous pain.	0.022	−0.033	0.041	0.012	0.009	**0.899**
No.3 I know what causes pain to become worse.	−0.086	0.053	0.075	−0.017	0.000	**0.690**
No.1 I know the times when pain is likely to appear.	0.263	0.011	−0.159	0.057	−0.053	**0.588**

^a)^ Opioid Self-management Scale for Advanced cancer patients with Pain

**Fig. 1:**
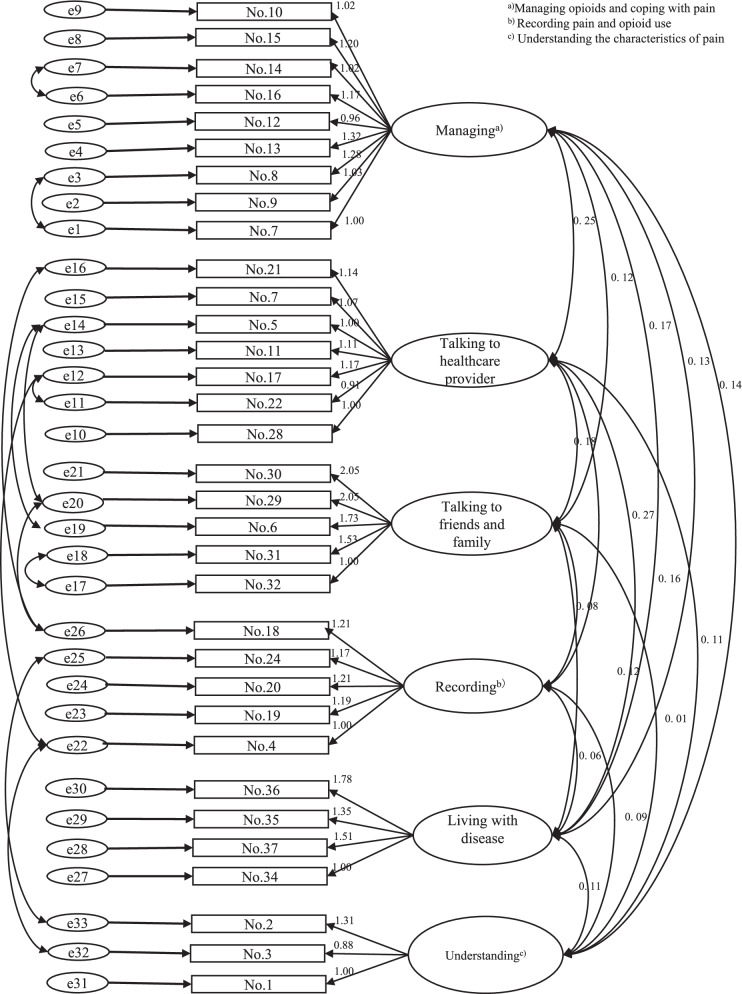
Standardized path diagram of the OSSA confirmatory factor analysis

Table 4 shows the results of the multitrait scaling analysis. The discriminant correlation coefficient range was − 0.02 to 0.64. The convergent correlation coefficient range was 0.54–0.96, and the scaling success rate was 100% for all items (Fig 1).

**Table 4:** Distinctive validity and convergent validity of the OSSA^a)^

Subscale	No. of items	Discriminant validity (range of correlation coefficients)^b)^	Convergent validity (range of correlation coefficients)^c)^	Scaling success (%)^d)^
Managing opioids and coping with pain	9	0.16–0.56	0.65–0.78	126/126(100%)
Talking to healthcare provider	7	0.01–0.64	0.73–0.93	84/84(100%)
Talking to friends and family	5	−0.02 – 0.50	0.62–0.96	50/50(100%)
Recording pain and opioid use	5	0.00–0.34	0.54–0.89	50/50(100%)
Living with the disease	4	0.06–0.52	0.73–0.94	36/36(100%)
Understanding the characteristics of pain	3	0.09–0.35	0.56–0.90	24/24(100%)

^a)^ Opioid Self-management Scale for Advanced cancer patients with Pain

^b)^Pearson’s correlation coefficient between the score for each item and the score for each domain excluding that item

^c)^Pearson’s correlation coefficient between the score for each item and the scores for the domains to which that item does not belong

^d)^ Number of correlation coefficients for which convergent correlation is higher than distinctive correlation/total number of correlation coefficients

The results of the confirmatory analysis are shown. The values were χ^2^ = 709.8 (*p <* 0.001, df = 467), goodness-of-fit index (GFI) = 0.78, adjusted goodness-of-fit index (AGFI) = 0.73, root mean squares of approximation (RMSEA) = 0.063, and comparative fit index (CFI) = 0.92.

Table 5 shows the relationships between the OSSA subscales and levels of pain severity. There was a significant negative correlation (*r =* − 0.21) between the total OSSA score and “*Average pain in 24 hours*,” and there was a significant positive correlation (*r =* 0.26) between the total OSSA score and “*Pain relief rate in 24 hours*.” Both of these correlation coefficients are “low” according to Guilford’s Rule of Thumb [30]. There were no significant correlations in pain association for the subscales “*Recording pain and opioid use*” and “*Understanding the characteristics of pain*.”

**Table 5:** Association between the OSSA^a)^ and pain

	Worst pain in 24 hours	Average pain in 24 hours	Pain relief rate in 24 hours	Interference with life by pain over 24 hours
Total OSSA^a)^ score	−0.08	−0.21^*^	0.26^**^	0.02
Managing opioids and coping with pain	−0.09	−0.22^*^	0.24^**^	0.08
Talking to healthcare provider	−0.12	−0.21^*^	0.14	0.02
Talking to friends and family	−0.11	−0.12^*^	0.15	0.04
Recording pain and opioid use	−0.03	−0.07	0.14	0.06
Living with the disease	−0.05	−0.13	0.23^**^	−0.11
Understanding the characteristics of pain	0.13	0.06	0.10	−0.07

Pearson’s correlation coefficient

^**^ *p <* 0.01 ^*^ *p <* 0.05

^a)^ Opioid Self-management Scale for Advanced cancer patients with Pain

#### Concurrent validity

Table 6 shows the analysis of the concurrent validity of the OSSA and SCAQ. The range of the correlation coefficients between the total OSSA score and the SCAQ total score and its 4 subscales was 0.59–0.75, indicating a correlation with moderate to very high significance. The OSSA subscales “*Recording pain and opioid use*” and “*Understanding the characteristics of pain*” were not correlated with either of the SCAQ subscales “*Ability to adjust one’s own physical condition based on personal weaknesses*” or “*Ability to concentrate one’s attention on self-care*.”

**Table 6:** Association between the OSSA^a)^ and the SCAQ^b)^

Item	Total SCAQ^b)^score	Ability to perform self-care operations	Ability to adjust one’s own physical condition based on personal weaknesses	Ability to concentrate one’s attention on self-care	Ability to receive valid support
Total OSSA^a)^ score	0.75^**^	0.69^**^	0.59^**^	0.61^**^	0.67^**^
Subscale 1: Managing opioids and coping with pain	0.60^**^	0.56^**^	0.48^**^	0.51^**^	0.48^**^
Subscale 2: Talking to healthcare provider	0.61^**^	0.49^**^	0.56^**^	0.55^**^	0.55^**^
Subscale 3: Talking to friends and family	0.67^**^	0.58^**^	0.45^**^	0.60^**^	0.68^**^
Subscale 4: Recording pain and opioid use	0.20^*^	0.18^*^	0.15	0.13	0.23^**^
Subscale 5: Living with the disease	0.71^**^	0.73^**^	0.57^**^	0.54^**^	0.54^**^
Subscale 6: Understanding the characteristics of pain	0.22^*^	0.26^**^	0.17	0.09	0.18^*^

Pearson’s correlation coefficient

^**^ *p <* 0.01 ^*^ *p <* 0.05

^a)^ Opioid Self-management Scale for Advanced cancer patients with Pain

^b)^ Self Care Agency Questionnaire

Table 7 shows the analysis of the concurrent validity of the OSSA and the Medication Adherence Scale. The range of the Pearson correlation coefficients between the total OSSA score and the Medication Adherence Scale total score and its four subscales was 0.32–0.62, indicating low-to-moderate significance. The OSSA subscale “*Recording pain and opioid use*” was not correlated with the Medication Adherence Scale subscales “*Medical compliance*” or “*Collaboration with healthcare providers*.” The OSSA subscale “*Living with the disease*” was not correlated with the Medication Adherence Scale subscale “*Medical compliance*.” The OSSA subscale “*Understanding the characteristics of pain*” was not correlated with the Medication Adherence Scale medication adherence scale subscales “*Medical compliance*” or “*Acceptance to take medication and how taking medication fits patient’s lifestyle*.”

**Table 7:** Association between the OSSA^a)^ and the Medication Adherence Scale

Item	Total Medication Adherence Scale score	Medication compliance	Collaboration with healthcare providers	Willingness to access and use information about medication	Acceptance to take medication and how taking medication fits patient’s lifestyle
Total OSSA^a)^ score	0.62^**^	0.32^**^	0.48^**^	0.56^**^	0.34^**^
Subscale 1: Managing opioids and coping with pain	0.62^**^	0.36^**^	0.43^**^	0.59^**^	0.34^**^
Subscale 2: Talking to healthcare provider	0.58^**^	0.25^**^	0.53^**^	0.52^**^	0.28^**^
Subscale 3: Talking to friends and family	0.36^**^	0.24^**^	0.29^**^	0.23^**^	0.25^**^
Subscale 4: Recording pain and opioid use	0.25^**^	0.13	0.10	0.25^**^	0.25^**^
Subscale 5: Living with the disease	0.35^**^	0.13	0.29^**^	0.30^**^	0.23^**^
Subscale 6: Understanding the characteristics of pain	0.18^*^	0.04	0.23^**^	0.23^**^	−0.08

Pearson’s correlation coefficient

^**^ *p <* 0.01 ^*^ *p <* 0.05

^a)^ Opioid Self-management Scale for Advanced cancer patients with Pain

Table 3 presents the reliability analysis. The overall Cronbach’s α coefficient of the OSSA was 0.93. The Cronbach’s α coefficients for the subscales had a 0.78–0.93 range. For the retest, we analyzed responses from 107/133 questionnaires (80.5%). The intraclass correlation coefficient (ICC) for the subscales had a 0.62–0.86 range.

## Discussion

The main finding of this study was that the OSSA, consisting of 33 items and six subscales, was “acceptable”. The sub-concepts included “*Managing opioids and coping with pain*,” “*Talking to a healthcare provider*,” “*Talking to friends and family*,” “*Recording pain and opioid use*,” “*Living with the disease*,” and “*Understanding the characteristics of pain*.” In this study, we extracted concepts similar to the Opioid-Taking Self-Efficacy Scale–Cancer (OTSES-CA) developed by Liang et al. [17], and the new concept of living with the disease. Pain is “an unpleasant sensory and emotional experience that occurs when some tissue damage actually occurs, when tissue damage is likely to occur, or when such tissue damage occurs” [31]. It has both psychological and physical aspects. Breivik et al. reported that 32% of patients with cancer pain reported that the pain makes them want to die [32]. Twycross noted that total pain consists of physical, mental, social, and spiritual aspects, and that the relief of a patient’s tension and diminished anxiety alleviates the perception of pain [33]. Factors that lead to refusal of opioid medication include the idea that pain is a sign that the cancer is getting worse and that there is a gradual development of tolerance to opioids [34, 35]. Therefore, it is important for patients with advanced cancer to recognize that opioids are essential to help them adapt and live with the disease.

The confirmatory factor analysis demonstrated that the OSSA had a sufficient level of fitness. The GFI and AGFI of the OSSA were low. We believe that this is due to the large number of included variables. The CFI (closeness to 1 indicates a better model) and RMSEA (0.05–0.08 is a reasonable fit) [27] verified that the level of fitness was good.

The concurrent validity analysis confirmed the expected correlations and non-correlations between the OSSA and the SCAQ or the Medication Adherence Scale. The correlations of the predicted SCAQ items with the newly developed OSSA indicated high concurrent validity; the correlations predicted by concurrent validity had moderate to very high correlation coefficients. Between the OSSA and SCAQ, there was no correlation between the OSSA “*Recording pain and opioid use*” and “*Understanding the characteristics of pain*” and SCAQ “*Ability to adjust one’s own physical condition based on personal weaknesses*” and “*Ability to concentrate one’s attention on self-care*.” This result indicates that the OSSA is a better tool for the self-management of opioid medications for pain relief.

The correlations of the predicted the Medication Adherence Scale items with the newly developed OSSA indicated high concurrent validity. This concurrent validity confirmed the predicted correlations: correlation coefficients between the OSSA and the Medication Adherence Scale were moderate for most items, although some items had low correlation coefficients. Between OSSA and the Medication Adherence Scale, there was no correlation between the OSSA subscales “*Recording pain and opioid use*,” “*Living with the disease*,” “*Understanding the characteristics of pain*,” and “*Medical compliance*” on the Medication Adherence Scale. This result indicates that since the Medication Adherence Scale is specialized for adherence, it does not contain items related to opioid-specific coping measures that can help improve the understanding of disease characteristics and measure the psychological adaptation to advanced cancer. As such, the OSSA demonstrated a high concurrent validity. Furthermore, examination of the discriminant validity, convergence validity, and scaling success rate in a multitrait scaling analysis confirmed that the OSSA has a constructive validity.

In the reliability analysis, the α coefficient, which is ideally ≥0.7 for use in research and ≥ 0.9 for clinical use [36], was 0.93 for the OSSA, suggesting that the OSSA is sufficiently consistent. In retesting, an ICC of 0.61–0.80 is considered substantial [37]. The ICC range in this study was 0.62–0.86, confirming the high reproducibility of the OSSA. The aforementioned tests confirmed the acceptable validity and reliability of the OSSA, suggesting a readiness for practical use.

The results of the hypothesis testing were consistent with the hypothesis that patients with high opioid self-management have significantly lower 24-hour average pain and a higher rate of pain relief. Furthermore, subscales concerning managing opioids and coping with pain, talking to healthcare providers, and talking to friends and family were associated with the alleviation of persistent pain. Our study supports the importance in alleviating pain for compliance to prescribed medication [17], symptom-coping efficacy [38], and reception of help from those around the patient [39].

In addition, *living with the disease* was associated with a significant increase in pain-relief rates. Patients with advanced cancer experience anxiety about an uncertain future and death, which can exacerbates symptoms such as depression, mental distress, existential distress, and social distress [40]. Pain is an unpleasant sensory and emotional experience [2]; since emotions are also involved, relief of psychiatric symptoms is effective for pain relief. Therefore, cognitive behavioral therapy and relaxation are recommended [41]. Furthermore, having a positive mentality even in advanced cancer stages can reduce anxiety and depression, leading to a better QOL [42]. These findings suggest that care for mental and existential distress is important in treatment using opioids.

In this study, the terms “*Recording pain and opioid use*” and “*Understanding the characteristics of pain*” were not significant in pain relief. This may be because the average duration of analgesic opioid use among the participants in this study was as long as 9 months; thus, they may have already understood the characteristics of pain and been able to tolerate it. However, the effect of recording pain and opioid use in a diary has been reported [43–45]. For a better QOL, patients can manage opioid use better when they understand the characteristics of their pain.

The OSSA will help to clarify the self-management abilities of patients in a clinical context and determine the need for nursing care according to 1) whether an intervention is required for the patient’s cognitive function, 2) whether a mental intervention is needed, 3) whether intervention for adjusting medication is needed, or 4) whether adjustments are needed for the patient’s living environment, based on the evidence of cancer pain alleviation.

Our study had some limitations. First, the generalizability of the results is very limited because the studied population included only outpatients. We plan to conduct a trial on hospitalized patients with advanced cancer. Second, the majority of the patients were using oxycodone (76%); therefore, our results are applicable mostly for outpatients who are using oxycodone. Since some drugs can be administered orally, intravenously, or as paste medicine, we plan to conduct a study on these drugs as well. Third, we need to establish a cut-off value in the future for the practical application of OSSA. Finally, although 33 items are effective for use in research, it is not realistic for patients to answer 33 questions in a real-world clinical setting. In the future, it will be necessary to develop a shortened version that is more applicable for patients in clinical settings.

## Conclusions

This study demonstrated that the OSSA has acceptable validity and reliability, and the results suggested that a higher self-management ability leads to greater pain relief. The original 40-item scale was reduced to 37 items through a Phase 1 content validity study, and two of the 37 items were revised in a Phase 2 face validity study. Finally, 33 items were developed in the final version by Phase 3, which evaluated the acceptable construct validity and criterion-related validity and reliability of the OSSA. The OSSA can be considered effective for use in research; however, a shortened version should be prepared for realistic and practical clinical use.

## Supplementary Information

**Additional file 1.**

## Data Availability

The datasets used and/or analyzed during the current study are available from the corresponding author on reasonable request.

## Abbreviations

AGFI: Adjusted goodness of fit index
CFI: Comparative fit index
CVR: Content validity ratio
GFI: Goodness of fit index
ICC: Intraclass correlation coefficient
KMO: Kaiser–Meyer–Olkin
OSSA: Opioid self-management scale for patients with advanced cancer with pain
OTSES-CA: Opioid-taking self-efficacy scale-cancer
PS: Performance status
QOL: Quality of life
RMSEA: Root mean squares of approximation
SCAQ: Self-care Agency Questionnaire
WHO: World Health Organization
